# Simultaneous Method for Selected PBDEs and HBCDDs in Foodstuffs Using Gas Chromatography—Tandem Mass Spectrometry and Liquid Chromatography—Tandem Mass Spectrometry

**DOI:** 10.3390/toxics11010015

**Published:** 2022-12-24

**Authors:** Eva Lipičar, Danijela Fras, Nino Javernik, Helena Prosen

**Affiliations:** 1 National Laboratory of Health, Environment and Food, Centre for Chemical Analysis of Food, Water and Other Environmental Samples, Prvomajska ulica 1, 2000 Maribor, Slovenia; 2 Faculty of Chemistry and Chemical Technology, University of Ljubljana, Večna pot 113, 1000 Ljubljana, Slovenia

**Keywords:** brominated flame retardants, hexabromocyclododecane—HBCDD, polybrominated diphenyl ethers—PBDE, POPs, GC-MS/MS, LC-MS/MS, ultrasound-assisted extraction, clean-up, validation

## Abstract

Flame retardants are added to consumer products to retard the ignition of combustible materials. Technical mixtures of polybrominated diphenyl ethers (PBDEs) and hexabromocyclododecane (HBCDD) were massively used for several decades. They are bioaccumulative, persistent, and have adverse effects on organisms. Recognised as persistent organic pollutants, they are banned almost worldwide. Food is the principal source of human exposure. Yet, no maximum residue limits for food have been established in the EU. Nevertheless, monitoring of specific congeners is recommended. Simultaneous analysis of HBCDDs and PBDEs is rarely encountered, especially including BDE-209, as this thermally unstable congener is particularly challenging for analysis. We have developed a method for the simultaneous determination of all relevant PBDEs and HBCDDs recommended for monitoring by the EU. In the method, single sample preparation is used for different types of foodstuffs, applying ultrasound-assisted extraction, clean-up by gel permeation, and adsorption chromatography. Analyses were performed on the same extract, first by GC-MS/MS(EI) method for PBDEs and followed by LC-MS/MS(ESI) method for HBCDDs. The analytical method was validated on a blank sample of milk formula at 2–3 fortification levels, including recommended LOQ level of 0.01 µg/kg wet weight. Satisfactory accuracy with recoveries 85–119%, intra-day precision (1.5–11.3%), and inter-day precision (4.3–18.4%) was obtained. The method ensures LOQs that are compliant with the EU recommendations for all PBDEs and HBCDDs, including BDE-209. Method applicability was further confirmed on proficiency testing samples of baby food, fish, and citrus.

## 1. Introduction

Flame retardants are chemicals added to different consumer products to inhibit possible combustion in the event of a fire, consequently reducing product flammability and improving product safety. To date, more than 175 flame retardants have been commercially produced, among which brominated flame retardants (BFR) are the most widely used due to being very effective and relatively cheap [1]. They are incorporated into electronics, textiles, plastics, and into building materials. Some of the products contain even up to 30% (*w/w*) of BFR compounds [2]. Mostly present as additives, they lack covalent bonding to the material and easily leach into the environment during usage, recycling, and disposal [3]. Their lipophilic character results in high affinity to adipose tissue; therefore, they bioaccumulate and biomagnify along the food chain and have been detected in numerous environmental and biological samples. They are also very persistent in the environment [1,2,3,4,5].

The use of brominated flame retardants started in the 1970s [1]. In the past, the most widely used flame retardants were hexabromocyclododecane (HBCDD) and mixtures of polybrominated diphenyl ethers (PBDEs), commercially known as penta-, octa- and decaBDE mixture, named after the degree of bromination of the main ingredient [6]. PBDEs are diphenyl ethers with different degrees of bromination, ranging from 1 to 10, which results in 209 possible congeners. HBCDD also exists in different isomers, with alfa, beta, and gamma-HBCDD being the predominant diastereomers in the commercial technical mixture HBCDD [6]. However, research has shown that PBDEs and HBCDD cause adverse effects on humans and ecosystems, with endocrine disrupting potential, neurodevelopmental effects, and toxicity to reproductive systems being of the highest concern [7,8]. All these facts led to their inclusion as persistent organic pollutants (POPs) in the annexes of the Stockholm Convention from 2009 to 2017 [9]. To date, 185 countries, European Union (EU) countries included, have signed the agreement to restrict and eliminate the production and use of PBDEs and HBCDD [10].

In general, the main exposure route to PBDEs and HBCDD is dietary intake [6]. Nevertheless, no regulations for the control of PBDEs and HBCDD in food and feed, such as MRLs, have yet been established by the EU authorities. However, a Commission Recommendation (2014/118/EU) on monitoring of traces of BFRs in food and feed was issued in 2014, specifying congeners of PBDEs of interest (BDE-28, 47, 49, 99, 100, 138, 153, 154, 183, and 209), recommending diastereomer specific determination of HBCDDs (α-, β-, and γ-) and setting different criteria on analytical methodology, including LOQ, which should be lower or equal to 0.01 µg/kg wet weight [11].

Considerable research has already been performed in this area resulting in several published methods. The separation technique of choice for the analysis of PBDEs is gas chromatography (GC), which allows for the lowest detection limits. Mass spectrometry (MS), with its superior selectivity, is the most frequent detection technique in the determination of PBDEs. Authors report coupling GC with different types of mass analysers and utilising both electronic (EI) and chemical (CI) ionisation modes [12,13,14,15,16,17,18]. Nevertheless, some authors have demonstrated fitness for the purpose of high-performance liquid chromatography (LC) for analysis of PBDEs, using mass spectrometry with atmospheric pressure chemical ionisation (APCI) [19] or atmospheric pressure photoionisation (APPI) [20,21,22], while electrospray ionisation (ESI) has proven to be ineffective for analysis of PBDEs, owing to poor ionisation of nonpolar and aromatic compounds [19,20]. Thermal interconversions of HBCDDs occur at temperatures above 160 °C; therefore, for speciated determination of HBCDD isomers, which is a requisite stated in the 2014/118/EU, the only appropriate chromatographic technique is liquid chromatography [23]. Coupled with tandem mass spectrometry (MS/MS), it provides an instrument for the analysis of HBCDDs at sub-ppb levels, which is already well documented [6,15,18,24,25,26,27,28].

Despite the substantial number of publications describing the methodology for the analysis of PBDEs in food and feed, only a handful of methods deal with the simultaneous analysis of HBCDDs and PBDEs [6,12,15,16,18,20,25,29,30,31,32,33,34]. Furthermore, only a few include BDE-209 [6,12,15,16,18,20,32,34], the main component of decaBDE commercial mixture, in the method for analysis of PBDEs. BDE-209 was the last PBDE put under regulation by the Stockholm Convention in 2017, and owing to its high boiling point and thermal instability, achieving the LOQ recommended by the 2014/118/EU is particularly challenging [2,6,29]. Authors reporting such low LOQs suggest using GC coupled to high-resolution mass spectrometry (HRMS) [12,13,14,16,18]. This sophisticated technique is undoubtedly fit for the purpose. However, the high cost of the instrument and the requirements of well-trained personal makes this instrument less available, especially in developing countries. An interesting alternative is tandem mass spectrometry (MS/MS), which is also highly selective and sensitive, while its price is substantially lower than HRMS and is less operationally demanding. There have been a few recent papers utilising atmospheric pressure chemical ionisation (APCI) GC-MS/MS for the analysis of PBDEs [35,36]. This soft ionisation technique makes quantitation at an ultra-trace level less challenging also for the PBDEs with higher bromination degrees, achieving very low quantification levels. However, this instrument is rarely found in laboratories, while GC-MS/MS with electron ionisation (EI) mode is much more common, owing to its robustness and versatility.

To address the issue of a simple method using commonly available instrumentation, we have developed a method for simultaneous determination of all nine relevant PBDE congeners (BDE-28, 47, 49, 99, 100, 153, 154, 183, and 209) and HBCDD isomers (α-, β-, and γ-) in different types of food and feed, using single sample preparation method and dual detection, GC-MS/MS(EI) for PBDEs and LC-MS/MS(ESI) for HBCDDs. To the best of our knowledge, only one method with single sample preparation for both HBCDDs and PBDEs, including BDE-209, and using the alternative tandem mass spectrometry in EI mode for PBDE detection has been published, but the detection level for BDE-209 was not sufficient to meet the 2014/118/EU recommendation [6,11]. The performance of the developed method was evaluated with fortified samples of uncontaminated infant milk formula and further assessed using archived proficiency testing samples.

## 2. Materials and Methods

### 2.1. Reagents and Sorbents

All solvents, reagents, and chromatographic sorbents used were analytical grades or above and were of suitable purity for residual analysis. Dichloromethane, *n*-hexane, and toluene were purchased from Honeywell (Seelze, Germany). Methanol, acetone, acetonitrile, and anhydrous sodium sulfate (Na_2_SO_4_) were supplied by J.T. Baker (Deventer, the Netherlands). Potassium hydroxide (86%) was obtained from Fisher Chemicals (Pittsburgh, PA, USA). Silica gel (60 Å, 35–60 mesh) and Carbopack C (60/80 mesh) were purchased from Supelco (Bellefonte, PA, USA), sulfuric acid (96%) from Carlo Erba (Rodano, Milano, Italy) and BioBeads S-X3 (200–400 mesh) from Bio-rad (Munich, Germany). All chromatographic sorbents used (acidic silica, potassium silicate, silica and Na_2_SO_4_) were prepared as stated elsewhere [37].

### 2.2. Standards

All standards used were 97.0% purity or higher.

Reference standard solutions of BDE-209 (50 µg/mL) and BDE-49 (50 µg/mL) were purchased from Cambridge Isotope Laboratories (Andover, MA, USA). Mixture of ^13^C_12_-labelled α-, β- and γ-HBCDD at 50 µg/mL and commercial mixtures of PBDEs at 1 ng/mL (10 ng/mL for BDE-209), containing ^13^C_12_-labelled (“Method 1614 labelled surrogate stock solution”) and native BDE-28, 47, 99, 100, 153, 154, 183, and BDE-209 (“Method 1614 native par stock solution”) as well as calibration solutions containing native (at 1, 5, 50, 500 and 2500 ng/mL) and ^13^C_12_-labelled analytes (at 100 ng/mL) (“Method 1614 calibration solutions CS1-CS5”) and standard mixture, containing 28 native PBDEs at 5–25 ng/mL and 12 ^13^C_12_-labelled PBDEs at 100–500 ng/mL (“ROHS PBDE calibration solution CS2”), used for evaluating selectivity of the developed method, were also obtained from the same supplier. Analytical standards α-, β- and γ-HBCDD were supplied by Sigma-Aldrich (Steinheim, Germany).

Calibration solutions “Method 1614 calibration solutions CS1-CS5”, containing native and labeled standards (1–2500 ng/mL) were used for quantifying BDE-28, 47, 99, 100, 153, 154 and BDE-183. Calibration solutions for BDE-209 and BDE-49 were prepared separately at the same concentration levels by combining and diluting certified standards of BDE-209 or BDE-49 and Method 1614 labelled surrogate stock solution so that same concentration levels were achieved.

Individual stock solutions of α-, β- and γ-HBCDD at 1 g/L were prepared in methanol. Stock solutions and labelled HBCDDs stock solutions were combined and diluted in methanol to prepare standard calibration solutions at 0.2, 0.5, 1, 2, 5, 10, 20 and 80 ng/mL of native HBCDDs and 60 ng/mL of labelled HBCDDs. The vials containing calibration standards were carefully concentrated under a gentle stream of nitrogen to dryness and re-dissolved in methanol/water (75:25, *v/v*).

For fortification experiments, standard solutions at 10 and 100 ng/mL, containing native PBDEs and HBCDDs, were prepared by combining and diluting native PBDEs stock solution and BDE-49 reference standard and by combining and diluting individual stock solutions of α-, β- and γ-HBCDD. For fortification experiments of BDE-209 at 0.01 µg/kg, separate standard solution at 10 ng/mL was prepared by diluting reference standard BDE-209.

For instrumental method development, individual standard solutions of α-, β- and γ-HBCDD at 50 ng/mL and standard solution of BDE-209 at 50, 100, 500, and 5000 ng/mL were prepared by making appropriate dilutions of the stock solution.

All standards were stored in amber containers and kept at 4 °C.

### 2.3. Test Samples

During screening of different foodstuffs and feed, a sample of infant formula (ecological cow milk) with very low levels of contamination (<0.001 µg/kg) was traced. This sample enabled spiking at the target LOQ and was therefore used in our fortification experiments. For further assessment of the trueness of the proposed method and its applicability to different food and feed matrices, test materials from previous proficiency testing (PT) exercises organised by the European Union Reference Laboratory for halogenated persistent pollutants (EURL-POPs) in feed and food, were used. Performance of the method was assessed on three different food and feed matrices: baby food, composed of pork, pumpkin, and vegetable oil (BF-2101-BF), fish fillet (2001-Fl), and dried citrus pulp (2105-DCP).

### 2.4. Sample Treatment

The extracts were prepared following the modified EPA 1614 standard method [37].

10 g of homogenised sample was weighted in a 50 mL PP centrifuge tube (or 100 mL Erlenmeyer flask in case of wet samples) and spiked with 1 ng of labelled PBDEs (10 ng of labelled BDE-209) and 12 ng of labelled HBCDDs. After 30 min equilibration time, a sufficient amount of anhydrous Na_2_SO_4_ was added to form a free-flowing powder. After 1 h drying time, 30 mL of dichloromethane:hexane (1:1, *v/v*) was added to the mixture. The mixture was vortexed for 1 min and ultrasonically extracted (UAE) for 1 h at 40 °C in an ultrasonic bath (Sonis 4, Iskra PIO, Šentjernej, Slovenia). After centrifugation at room temperature for 3 min at 2500 rpm, the supernatant was carefully decanted into a distillation bulb, and the extraction process was repeated.

The combined crude extract was purified using gel permeation and adsorption chromatography. First purification step was performed by using a multi-layer silica column packed with (bottom to top): Na_2_SO_4_ (2 g), neutral silica (1 g), potassium silicate (1.5 g), neutral silica (1 g), acidic silica (35% *w/w*, 25 g), neutral silica (1 g) and Na_2_SO_4_ (2 g). The combined extract, without prior concentrating step, was transferred to the top of the prepared column, and the analytes were eluted with additional 50 mL of dichloromethane:hexane (1:1, *v/v*). The eluate was concentrated using rotary evaporator to about 100 µL, quantitatively transferred to a vial and further concentrated to incipient dryness under a gentle nitrogen stream. The residue was re-dissolved in 300 µL dichloromethane:hexane (1:1, *v/v*) and put on an autosampler of an HPLC (1100/1200 Series, Agilent Technologies, Santa Clara, CA, USA) consisting of quaternary HPLC pump, degasser, fraction collector and two valves. The extract was injected into a GPC column (Bio Beads S-X3, 70 g) with a flow of dichloromethane:hexane (1:1, *v/v*) set at 2.5 mL/min. The compounds in the collection fraction of 64–100 mL were further isolated by fluxing through active carbon (Carbopack C, 200 mg). The analytes were eluted from carbon with additional 45 mL of hexane.

The purified extract was concentrated to about 100 µL, transferred to a vial equipped with an insert and further concentrated to dryness under a gentle stream of nitrogen, and the dry residue was dissolved in 20 µL of toluene.

The extracts were analysed for PBDEs with GC-MS/MS (EI). After the analysis, they were carefully concentrated to dryness, and the dry residue was re-dissolved in 200 µL of methanol:water (75:25, *v/v*) and analysed with LC-MS/MS (ESI) for HBCDD on the same day.

During validation of the method, blank infant milk formula was spiked at two different levels (at 0.01 µg/kg and 0.3 µg/kg) for PBDE 28–183 and HBCDDs and at three different levels for BDE-209 (at 0.01 µg/kg, 0.1 µg/kg, and 0.3 µg/kg). The fortification experiments on each level were performed in sextuplicate using 50 mL PP centrifuge tubes. To assess within-laboratory reproducibility, all experiments were repeated at least a week later by a different technician. The methods’ performance was further evaluated by analysing archived PT samples of fish fillet, dried citrus pulp, and composite baby food.

To optimise the extraction method, the sample of fish fillet was analysed in two modifications. First in six parallels, using PP centrifuge tubes and 20 g of anhydrous Na_2_SO_4_, and second by employing 100 mL Erlenmeyer flask, facilitating bigger amount of the desiccant (40 g).

### 2.5. Instrumental Analysis

#### 2.5.1. GC-MS/MS (EI) Method

Separation and detection of PBDEs were carried out by a 7890B gas chromatograph (Agilent Technologies), equipped with a 7693 autosampler (Agilent Technologies) and coupled to a 7010B triple-quadrupole mass spectrometer (Agilent Technologies). In the optimised method, 5 µL of standards and extracts were injected using a multi-mode inlet (MMI) set on solvent vent mode. Vent flow and pressure were set at 100 mL/min and 5 psi, vent time was 0.25 min, and inlet was switched to purge mode at 2.62 min (with a purge flow of 60 mL/min). Inlet initial temperature was set at 70 °C (0.25 min), followed by a quick ramp of 600 °C/min to 325 °C. PBDEs were separated on a 20 m × 0.18 mm I.D. ZB-Semivolatiles (Phenomenex, Macclesfield, Cheshire, UK) analytical column with 0.18 µm film thickness and helium as the carrier gas at constant flow of 1.4 mL/min. GC oven temperature programme was as follows: 70 °C (1.25 min), 20 °C/min to 240 °C and 50 °C/min to 320 °C (23 min).

Mass spectrometer was operated in electron ionisation mode with ionisation energy set at 70 eV. Ion source, transfer line and both quadrupoles were maintained at 280 °C, 300 °C, and 150 °C, respectively. Helium (2.5 mL/min) was used as quench gas, and nitrogen (1.5 mL/min) as collision gas.

Quantitation of PBDEs was performed using five-point calibration curves (four-point for BDE-49) in multiple reaction monitoring (MRM) mode, whereas selected ion monitoring (SIM) was used for analysis of BDE-209. Two specific transitions from the same cluster were monitored for each congener BDE-28 to BDE-183 and its corresponding internal standard (IS)—one quantitative and the other confirmative, shown in Table 1. For BDE-209 and ^13^C_12_-BDE-209 three and two *m/z* were monitored, respectively.

Resolution of the quadrupoles was set to unit (0.7 Da). Masshunter (Agilent Technologies) was used to control the system and process the data.

#### 2.5.2. LC-MS/MS(ESI)

Analyses of HBCDDs were performed on a Chronet Symbiosys Plus HPLC system (Axel Semrau, Sprockhövel, Germany) coupled to Sciex 6500+ triple quadrupole mass spectrometer (AB Sciex, Darmstadt, Germany). The mass spectrometer was operated in negative electrospray ionisation mode.

During method development, two analytical columns were tested: Phenomenex Luna PFP (150 × 2 mm, 3 µm particles) and Luna C18 (150 × 2 mm, 3 µm particles), held at 30 and 40 °C. Mobile phase flow was set at 0.2 mL/min. Analytes were separated using binary gradient system consisting of methanol:water (75:25, *v/v*) (A) and acetonitrile (B). Initial composition 80% A was held for 1.00 min, followed by linear decrease in the next 1.00 min to 45% A, held at 45% A for 9.0 min and returned to initial conditions in 0.1 min. Injection volume was 70 µL.

In the optimised method, temperature of the ion source was kept at 300 °C, ion spray voltage was −4500 V, whereas optimised curtain, nebulising, drying and collision gas were 35.0, 35.0, 50.0 and 10.0 psig N_2_, respectively.

For native HBCDDs, two transitions per parent *m/z* were monitored, while only one transition was monitored for their labelled analogues. Selected MRM transitions and optimised parameters are presented in Table 2.

### 2.6. Quality Control

Due to UV lability of PBDEs, all standards and extracts were kept in amber glass or wrapped in aluminium foil. To improve reliability of the analyses, isotopically labelled analogues were used for all the analytes except BDE-49.

To assure reliable identification, two transitions (three ions for BDE-209) were monitored for every analyte, and the deviation of the ratios between both transitions (ions for BDE-209) were within 20% of the value obtained in the reference standard, analysed in the same sequence.

To control possible contamination, instrumental and procedural blanks were performed in every batch. The procedural blank was processed through the entire analytical procedure along with the samples. Batch was accepted when procedural blanks were less than 20% of the LOQ or the contribution to the result was less than 5%. The results were not corrected for blanks.

In every batch, the instrument was either calibrated or the calibration was checked before, and in both cases after the injection of extracts, by at least one reference standard. The acceptance criteria were set at 20%.

## 3. Results and Discussion

### 3.1. GC-MS/MS Method Development

Method for analysis of BDE-28 to BDE-183 in water and biota samples, routinely used in our laboratory in the past years, served as the starting point for the method development of GC-MS/MS method for analysis of PBDEs in food and feed. In the previous method, 2 µL of the extract was injected on DB-5MS UI (30 m × 0.25 mm × 0.25 µm) column in cold splitless injection mode. With instrumental LOQs for BDE-28 to BDE-154 below 1 ng/mL, this method would be suitable also for the analysis of PBDEs in food and feed, considering the target LOQ and sample amount. However, according to the 2014/118/EU recommendation, BDE-209 is to be included in the method for PBDEs in food and feed as well. To gain insight into the response of BDE-209 using our already established method, a standard solution of BDE-209 at 5 µg/mL was injected in full scan mode. No peak for BDE-209 was observed. This was consistent with reports by other authors that shorter column, preferably with low stationary phase film thickness, should be employed for analysis of highly brominated BDEs, such as BDE-209, to minimise column residence time and avoid analyte degradation [1,2,4,13,14]. From this perspective, the Phenomenex Zebron ZB-Semivolatiles column (20 m × 0.18 mm × 0.18 µm) was installed, and the experiment was repeated, following the oven and inlet temperature programme of Phenomenex application note [38]. Using this column, a peak corresponding to BDE-209 was observed at 24.1 min with an S/N ratio of 10. This column was thus selected for further optimisation.

To maximise the response for BDE-209, a large volume injection of 5 µL was considered. The MMI inlet used was therefore switched to the solvent vent mode. Solvent vent parameters were determined by using a solvent elimination wizard integrated with Masshunter software. Injection volume, solvent type, and the boiling point for the first eluting analyte (300 °C) were supplied, whereas the inlet temperature was set to 70 °C, as in our previous method. A vent flow of 100 mL/min at 5 psi for 0.12 min and an injection rate of 41 µL/min were suggested by the software. Using these parameters, 5 µL of BDE-209 standard solution at 500 ng/mL was injected into the system in MRM mode (transition *m/z* 799.4 to 639.5 at 45 eV [39]). A peak with S/N 50 was observed. However, injecting 5 µL of BDE-209 at 100 ng/mL gave a signal with poor intensity (S/N < 10). With target LOQ 0.01 µg/kg w.w. in food and feed (corresponding to 5 ng/mL in final extract) set for all the BDEs, including BDE-209, the method did not reach adequate LOQs. For this reason, the effects of changing the initial inlet temperature and applying constant flows of 1.2, 1.4, and 1.6 mL/min were investigated. Setting the initial inlet temperature to 100 °C resulted in a significantly lower response for BDE-209. Due to the use of compressed air for cooling the inlet between analyses, using lower temperatures would not be practical and was therefore not tested. Compared to the constant flow of 1.2 and 1.6 mL/min, applying 1.4 mL/min gave the best results. The response for BDE-209, compared to the response using ramped flow, increased approx. 20-fold.

In the next step, a standard solution containing all relevant PBDEs at 20 ng/mL (200 ng/mL for BDE-209) was injected to evaluate solvent vent mode conditions on early eluting BDEs. Peak humps were observed before target peaks, affecting all peaks from the first eluted BDE-28 until hexa-BDEs, indicating the need to further optimise the solvent vent parameters. In this regard, the vent time was prolonged to 0.25 min, which greatly reduced the humping effect and improved the peak symmetry. To evaluate the detection capability of the optimised method for BDE-209, 5 µL at 50 ng/mL was injected, resulting in an S/N of 10. Furthermore, a full scan (*m/z* 50–1000) of a standard solution of BDE-209 at 5 µg/mL was acquired. The intensity for the cluster of molecular ions was quite scarce due to EI at 70 eV used. A cluster corresponding to the loss of two bromine atoms showed the highest intensities in the *m/z* range of 450 to 1000. Optimised GC method was evaluated again with the selected ion monitoring (SIM) detection mode, using the highest *m/z* from the octa-BDE cluster (*m/z* 799.5). The response for BDE-209 in SIM mode compared to MRM mode was improved by a factor of 25, as also reported by other authors [6,40]. The detection was tested on the first quadrupole as well as on the second, the latter yielding a 20% higher response. To improve the certainty of identification by comparison of the area ratios in extracts and reference standards, two additional most abundant ions from the same cluster were selected for monitoring. Finally, 1 ng/mL of BDE-209 was injected in three repetitions, resulting in 11% RSD (corrected by isotope dilution) and indicating that the method was suitable to proceed to the validation step.

Precursor ions and product ions for MRM monitoring of BDE-28 to BDE-183 and their corresponding labelled analogues are already described elsewhere [36]. Collision energies for these transitions were optimised in the range of 0–60 eV, using a 5 eV step between acquisitions, followed by fine-tuning using ±1 eV step. The collision energies resulting in the highest responses were selected. Data acquisition was optimised to achieve at least ten points over each peak.

Analytes were baseline separated using the Phenomenex application note method [38]. Chromatograms of a standard solution of PBDEs at 1 ng/mL are shown in Suppl. Material, Figures S1 and S2. Before proceeding to the validation study, the efficiency of the separation was further assessed on BDE 49/71 pair, which is used as a criterion in EPA 1614 method [37]. Using the developed method, a standard commercial mixture containing a broader range of PBDEs, including BDE-49 and -71, was analysed. With 19% resolution, it was well within the criterion set in EPA 1614 method [37].

### 3.2. LC-MS/MS Method Development

To develop the LC-MS/MS method, 50 ng/mL of standard solutions containing single HBCDD isomers were injected into the MS/MS. The transitions, their collision energies (CE), collision cell exit (CXP), declustering (DP) and entrance potential (EP) were optimised using the Analyst optimiser program software. Dwell times were adjusted so that at least 10 points over peaks were achieved. The final parameters are given in Table 2.

A starting point for our LC method development was the paper by Hu et al. [28]. The first experiments were performed on Luna C18 analytical column with the same stationary phase as the Zorbax C18 (Agilent Technologies) used in the above-mentioned paper. The composition of the mobile phase was kept the same as well, while two different column temperatures were tested −30 and 40 °C, and signal intensities were compared. For α-HBCDD, no difference was observed in the peak height. In contrast, β- and γ-HBCDD showed an approx. 20% improvement when the analytical column was set at 40 °C. Based on our previous good experience in the analysis of other organic compounds using pentafluoro phenyl stationary phase analytical column, a parallel experiment was performed on Luna PFP (Phenomenex). Compared to the Luna C18 column, peaks of all HBCDDs were 100% more intensive. Luna PFP column at 40 °C was therefore selected to be used in our validation study. A chromatogram of a standard solution of α-, β-, and γ-HBCDD at 1 ng/mL is shown in Suppl. Material, Figure S3.

### 3.3. Optimisation of the Extraction and Clean-Up Method

#### 3.3.1. Extraction Method

**Figure 1:**
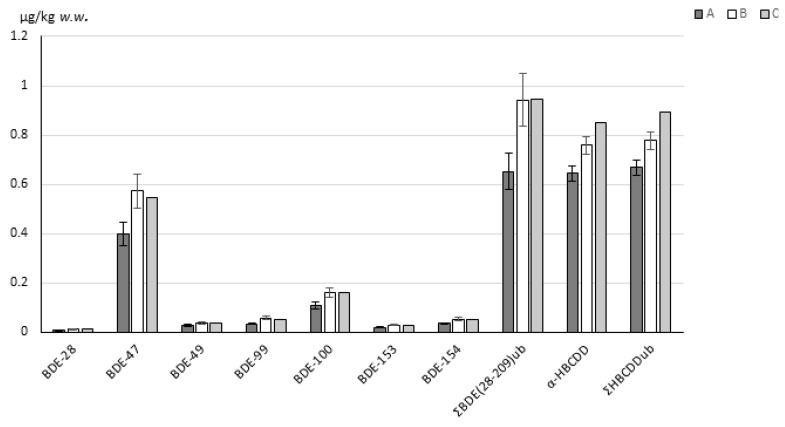
Comparison of the results for PT sample fish fillet, obtained with UAE and two different amounts of Na_2_SO_4_ with the assigned values: A: drying with 20 g Na_2_SO_4_, 60 mL dichloromethane:hexane (1:1, *v/v*), UAE; B: drying with 40 g Na_2_SO_4_, 60 mL dichloromethane:hexane (1:1, *v/v*), UAE; C: assigned values.

The proposed sample preparation is a modification of the procedure described in the standard EPA 1614A method [37] for the determination of PBDEs. In EPA 1614A, PBDEs are extracted from tissue with Soxhlet extraction. Owing to its high efficiency and simplicity, Soxhlet extraction is the traditional approach extraction-wise for ultra-trace analysis of POPs in complex matrices such as food and feed and is next to the accelerated solvent extraction most frequently used in the field of determination of BFRs [12,13,14,15,16,20,25,26,32,36]. However, this technique requires high amounts of solvents and long extraction times. To increase laboratory throughput, ultrasound-assisted extraction, reported by some authors as efficient for extraction of BDEs from food and biota matrices [31,41,42,43], was considered. In the proposed method, 10 g of the sample is homogenised using a sufficient amount of anhydrous Na_2_SO_4_. A total of 10 g was evaluated to be the minimum aliquot of sample used to achieve the desired LOQs, taking into account a minimal final volume of the extract (20 µL) and detection capabilities of the applied instrumental methods. First experiments during method development were performed on a fortified sample of blank infant milk formula, which was in powder form. The sample was mixed with a spoon (1–2 g) of anhydrous Na_2_SO_4_ before it was extracted two times using 30 mL of dichloromethane:hexane (1:1, *v/v*), increasing the mass transfer by vortexing the mixture and ultrasonication for 1 h at 40 °C. After centrifugation, the supernatant was decanted, and the crude extract proceeded to the clean-up stage. The extraction efficiency was further evaluated on an archived sample of dried citrus pulp, and satisfactory analytical recoveries (85–127%) were obtained. In the next step, the performance of the developed method was tested on a wet sample of fish fillet. The sample was dried with 20 g of anhydrous Na_2_SO_4_, reaching the maximum capacity of the PP tube used. The recoveries obtained were 65–79% (mean of 6 independent determinations, RSD 4.7–12.8%). The analysis of the same sample was repeated using a 100 mL Erlenmayer flask, which allowed for a higher 40 g addition of anhydrous Na_2_SO_4_ while keeping the same amount of extraction solvent. The sample was not centrifuged but was allowed to settle, and the extract was then carefully decanted into a fresh distillation bulb. The extraction was repeated once more. This time the recoveries of the combined two extractions ranged from 89–113%, indicating the importance of proper desiccation of the sample for efficient extraction. The comparison of the results obtained in both experiments, and the assigned values of the test material, is presented in Figure 1. The results obtained with the developed method and a properly desiccated sample of fish fillet were in excellent agreement with the assigned values, confirming the methods’ applicability to fish samples.

ΣBDE(28-209)_ub_ is defined as sum of BDE-28, 47, 49, 99, 100, 153, 154, 183 and BDE-209 at upper bound (contribution of each non-quantified congener to the sum parameter equals LOQ of each non-quantified congener)

Σ(HBCDD)_ub_ is defined as sum of α-, β- and γ-HBCDD at upper bound (contribution of each non-quantified congeners to the sum parameter equals LOQ of each non-quantified congener)

#### 3.3.2. Optimisation of the Clean-Up Procedure

The clean-up procedure is a modification of the clean-up method used in our laboratory for the analysis of polychlorinated dibenzo-*p*-dioxins/furans and polychlorinated biphenyls (PCBs), which follows the principles stated in the EPA 1613 and EPA 1668 [44,45]. Briefly, the bulk of fat is removed by adsorption chromatography on a multi-layer silica column consisting of H_2_SO_4_-impregnated silica gel (35%, *w/w*), neutral silica gel and potassium silicate. In the next step, the extract is concentrated and further cleaned with GPC and fractionation on carbon, dividing the purified extract into three fractions. A standard solution containing PBDEs and HBCDDs was processed by the proposed clean-up method. All collected fractions, as well as the waste, were analysed. BDE-28 to BDE-183 and HBCDDs were eluted from the GPC column in the 64–84 mL fraction, together with the non-planar PCBs. To include BDE-209, the collection of the fraction had to be extended to include the next 16 mL previously put into waste. In the next step, the performance of the optimised clean-up method was tested on a sample of blank infant formula fortified at 0.3 µg/kg. A fraction of 64–100 mL contained all PBDEs and HBCDDs, including BDE-209, whereas the waste and other fractions were found to be empty. To assess for potential losses of the analytes during the first clean-up step, elution with an additional 50 mL of dichloromethane:hexane (1:1, *v/v*) was performed. The losses were 3–5% for BDE-28 to BDE-183, 5–10% for HBCDDs and for the most challenging BDE-209 0.5%. In order to keep the extract free of possible interferences, the method proceeded to the validation step without increasing the elution solvent volume by 50 mL.

### 3.4. Method Validation

#### 3.4.1. Linearity

Isotope dilution was used for the calibration of all PBDEs except BDE-49, which was quantified using internal standard ^13^C_12_-BDE-47 with the same bromination degree. The relative responses of native compounds to their labelled analogues (for BDE-28, 47, 99, 100, 153, 154, 183, and BDE-209) and the response factor of BDE-49 to ^13^C_12_-BDE-47 were plotted against five concentration ratios (1, 5, 50, 500, and 2500 ng/mL). Eight-level calibration curves for α-, β- and γ-HBCDD (at 0.2, 0.5, 1, 2, 5, 10, 20, and 80 ng/mL) were constructed in the same manner. Calibration curves were weighted to 1/x, which improved the quality of analytical results at the lower range. Linearity was evaluated based on the coefficient of determination (r^2^) and bias of the relative response factors. The instrumental method showed excellent linearity over the entire range, with a correlation coefficient of 0.995 or greater, whereas the RSD of response factors over the entire range was less than 15% for all target compounds except for BDE-49. To meet the same criterion, the last calibration point was excluded for BDE-49, resulting in a narrower linear range of 1–500 ng/mL for this analyte.

#### 3.4.2. Limit of Quantification (LOQ)

The LOQ was defined as the fortification level that meets the criteria for precision, expressed as RSD (<20%) and accuracy (70–130%), set in the guidelines for the determination of BFRs in food and feed [46]. To identify blank food samples that would be used for confirming LOQs of the developed method, 15 samples of foodstuffs and feed (eggs, mussels, fish, baby food, baby milk formulas, milk, and meat) were screened for PBDEs and HBCDDs. Due to PBDEs and HBCDD being ubiquitously present, all samples contained at least one of the analytes at levels above 10% of target LOQ (0.01 µg/kg), except for one sample of infant formula (ecological cow milk). To confirm the low LOQs of the PBDEs and HBCDDs, this sample was fortified at 0.01 μg/kg, and the intra-day and inter-day precision (expressed as RSD) and trueness (recovery) were calculated. The results are presented in Table 3. Intra-day precision at the LOQ level was 3.7–11.3%, inter-day precision 6.9–13.1%, whereas the recoveries ranged from 85–106%, meeting all the required criteria.

#### 3.4.3. Trueness and Precision

To assess the repeatability (intra-day precision) and reproducibility (inter-day precision) of the developed method, blank infant milk formula was spiked with BDEs and HBCDDs at 0.01 and 0.3 μg/kg (0.01, 0.1, and 3 μg/kg for BDE-209) and analysed in six parallels. The experiment on each level was repeated once more under within-laboratory reproducibility conditions, at least a week later, using different batches of solvents and sorbents and analysed by a different technician. Chromatograms of extract of the spiked blank sample at 0.01 μg/kg (0.1 μg/kg for BDE-209) are shown in Suppl. Material, Figures S4–S6; and of the spiked blank sample with 0.01 μg/kg for BDE-209 in Figure S7.

Intra-day precision, expressed by the relative standard deviation (RSD%) of six independent determinations, inter-day precision, expressed by RSD% of 12 independent determinations under within-laboratory reproducibility conditions, and mean recoveries (corrected for isotope dilution) for all the determinations were calculated, and are presented in Table 3.

For 0.3 µg/kg fortification level, the intra-day precision for BDE-28 to BDE-183 and HBCDDs was found to be from 1.5% to 5.6%, whereas inter-day precision varied from 4.3% to 16.4%. The mean recoveries of all analytes ranged from 95 to 119%. For the matrix-spike level, equivalent to the target LOQ, no significant differences were observed compared to higher fortification levels. Recoveries were 85–106%, repeatability 3.7–11.3% and reproducibility 6.9–13.1%. For BDE-209, repeatability and reproducibility were assessed on three fortification levels. The average recovery throughout the range (*n* = 36) was 96%. Intra-day precision (1.6–7.1%) was slightly better than inter-day precision (7.2–18.4%). These values meet the requirements for trueness and precision set in the guidelines for the determination of BFRs in food and feed [46].

To demonstrate the applicability of the method to other samples apart from milk formula, archived PT test samples were analysed using the proposed method: baby food (composed of pumpkin, pork, and vegetable oil), fish fillet, and citrus pulp. Trueness was assessed by comparing obtained results to the assigned values given in the reports of the PT organisers [47,48,49]. The results are presented in Table 4.

As it can be seen from Table 4, trueness expressed as recoveries of the proposed method ranged from 85 to 127% for all the analysed matrices and analytes.

## 4. Conclusions

An analytical method based on GC-MS/MS (EI) and LC-MS/MS (ESI) was developed for the quantitative determination of three HBCDD isomers and nine relevant PBDEs, recommended by the 2014/118/EU, including BDE-209. Ultrasound-assisted extraction used in this study has been shown to be equally suited for the determination of PBDEs and HBCDDs as the traditional Soxhlet extraction. The method was validated on infant milk formula and is characterised by satisfactory precision (repeatability and reproducibility) and trueness, reaching adequate LOQs of 0.01 μg/kg wet weight. Additional experiments on archived proficiency testing samples confirmed the applicability of the method to other food and feed matrices, such as meat, vegetable, fruit and fish. The developed method is rather simple and is suitable for control laboratories with commonly found analytical equipment, such as a triple-quadrupole tandem mass spectrometer.

## Figures and Tables

**Table 1:** MRM transitions with optimised collision energies (CE) for BDE-28 to BDE-183 and selected SIM *m/z* for BDE-209.

	Quantitative Transition/ion	Confirmative Transition/ions	Abundance Ratio (%RSD) ^a^
BDE-28	407.8 → 248.0 (12 eV)	405.8 → 246.0 (11 eV)	0.97 (1.6)
13C12-BDE-28	417.8 → 258.0 (13 eV)	419.8 → 260.0 (8 eV)	0.65 (1.4)
BDE-47, BDE-49	485.7 → 325.8 (14 eV)	487.8 → 327.8 (12 eV)	0.45 (2.4), 0.46 (0.6)
13C12-BDE-47	497.7 → 337.8 (5 eV)	499.7 → 339.8 (15 eV)	1.00 (1.3)
BDE-99, BDE-100	565.6 → 405.8 (20 eV)	563.6 → 403.8 (9 eV)	0.63 (0.9), 0.61 (5.2)
13C12-BDE-99, 13C12-BDE-100	577.6 → 417.8 (17 eV)	575.6 → 415.8 (10 eV)	0.74 (2.2), 0.72 (0.5)
BDE-153, BDE-154	643.7 → 483.8 (17 eV)	645.7 → 485.8 (18 eV)	0.70 (7.2), 0.66 (1.7)
13C12-BDE-153, 13C12-BDE-154	655.7 → 495.8 (18 eV)	657.7 → 497.8 (15 eV)	0.58 (1.6), 0.56 (1.3)
BDE-183	723.4 → 563.6 (22 eV)	721.4 → 561.6 (15 eV)	0.93 (7.3)
13C12-BDE-183	733.4 → 573.6 (16 eV)	735.4 → 575.6 (12 eV)	0.77 (3.5)
BDE-209	799.5	797.5, 801.5	0.83 (2.4), 0.83 (6.1)
13C12-BDE-209	811.5	809.5	

^a^ Average abundance ratio and standard deviation, acquired for a typical calibration curve.

**Table 2:** Optimised detection parameters for HBCDDs.

	Quantitative Transition	Confirmative Transition	Abundance Ratio (%RSD) ^a^	Declustering Potential (V)	Entrance Potential (V)	Collision Cell Exit Potential (V)
α-HBCDD	640.5 → 78.9 (−72 V)	640.5 → 80.9 (−72 V)	0.93 (14)	−45	−10	−9
13C12-α-HBCDD	652.6 → 80.9 (−52 V)	-		−35	−10	−9
ß-HBCDD	640.5 → 80.9 (−58 V)	640.5 → 78.9 (−58 V)	1.04 (7.4)	−55	−10	−9
13C12-ß-HBCDD	652.6 → 80.9 (−52 V)	-		−35	−10	−9
γ-HBCDD	640.5 → 78.9 (−62 V)	640.5 → 80.9 (−62 V)	0.91 (13)	−40	−10	−9
13C12-γ-HBCDD	652.6 → 80.9 (−52 V)	-		−35	−10	−9

^a^ Average abundance ratio and standard deviation, acquired for a typical calibration curve.

**Table 3:** Intra-day (*n* = 6), inter-day precision (*n* = 12), and average recoveries (*n* = 12) of fortified infant milk formula.

Analyte	Spike Level (µg/kg)	Intra-Day Precision (%)	Inter-Day Precision (%)	Recovery (%)
BDE-28	0.01	4.0	10.9	102
	0.3	1.5	16.4	95
BDE-47	0.01	5.8	10.4	101
	0.3	3.1	4.3	112
BDE-49	0.01	4.9	8.0	106
	0.3	5.2	9.1	119
BDE-99	0.01	5.1	13.1	102
	0.3	3.3	12.8	96
BDE-100	0.01	4.3	12.6	105
	0.3	1.9	13.7	98
BDE-153	0.01	5.6	9.6	98
	0.3	1.8	11.3	97
BDE-154	0.01	4.6	11.4	105
	0.3	2.6	12.4	106
BDE-183	0.01	6.1	11.9	99
	0.3	3.2	11.1	102
BDE-209	0.01	7.1	8.3	105
	0.1	2.8	10.4	94
	3	1.6	18.4	88
α-HBCDD	0.01	11.3	10.7	96
	0.3	5.1	5.2	98
β-HBCDD	0.01	3.7	6.9	85
	0.3	5.6	5.3	99
γ-HBCDD	0.01	6.9	/ ^a^	85 ^b^
	0.3	2.7	5.9	106

^a^ For γ-HBCDD inter-day precision on level 0.01 µg/kg was not determined. ^b^ Recovery given is the average recovery of six determinations under repeatability conditions.

**Table 4:** Results of analysed PT samples and calculated trueness of the determinations.

	Dried Citrus Pulp		Fish Fillet		Baby Food	
	Result (µg/kg w.w.)	Trueness (%)	Result (µg/kg w.w.)	Trueness (%)	Result (µg/kg w.w.)	Trueness (%)
BDE-28	0.009	98	0.013	113	<LOQ (0.003)	(113)
BDE-47	0.120	101	0.573	104	0.251	117
BDE-49	0.014	113	0.037	100	<LOQ (0.006)	(119)
BDE-99	0.159	102	0.057	111	0.312	109
BDE-100	0.035	105	0.161	100	0.067	108
BDE-153	0.016	105	0.028	97	0.030	92
BDE-154	0.014	105	0.053	102	0.024	100
BDE-183	0.008	109	<LOQ	n.d.	0.040	113
BDE-209	0.598	85	0.009	n.d.	0.297	86
ΣBDE(28-209)_ub_	0.975	90	0.942	100	1.03	103
α-HBCDD	0.086	n.d.	0.758	89	0.0946	105
β-HBCDD	0.059	n.d.	<LOQ	n.d.	<LOQ	n.d.
γ-HBCDD	1.21	127	0.010	n.d.	0.019	n.d.
ΣHBCDD_ub_	1.35	127	0.778	87	0.124	n.d.

n.d. = assigned value was not given in the PT sample. ΣBDE(28-209)_ub_ is defined as sum of BDE-28, 47, 49, 99, 100, 153, 154, 183 and BDE-209 at upper bound (contribution of each non-quantified congener to the sum parameter equals LOQ of each non-quantified congener). Σ(HBCDD)_ub_ is defined as sum of α-, β- and γ-HBCDD at upper bound (contribution of each non-quantified congeners to the sum parameter equals LOQ of each non-quantified congener).

## Data Availability

The data presented in this study are available on request from the corresponding author.

## Supplementary Materials

The following supporting information can be downloaded at: https://www.mdpi.com/article/10.3390/toxics11010015/s1, Figure S1: Chromatogram of standard mixture of PBDEs (BDE-28 to BDE-183) at 1 ng/mL, MRM transitions; Figure S2: Chromatogram of standard solution of BDE-209 at 1 ng/mL, SIM 799.5 *m/z*; Figure S3: Chromatogram of standard mixture of α-HBCDD (A), β-HBCDD (B) and γ-HBCDD (C) at 1 ng/mL; Figure S4: GC-MS/MS (EI) chromatogram of extract of infant milk formula, fortified at 0.01 µg/kg of BDE-28 to BDE-183, 0.01 µg/kg of α-, β- and -γ HBCDD and 0.1 µg/kg of BDE-209, quantitative MRM transitions for BDE-28 to BDE-183; Figure S5: GC-MS/MS (EI) chromatogram of extract of infant milk formula, fortified at 0.01 µg/kg of BDE-28 to BDE-183, 0.01 µg/kg of α-,β- and -γ HBCDD and 0.1 µg/kg of BDE-209, SIM 799.5 *m/z* (BDE-209); Figure S6: LC-MS/MS (ESI) chromatogram of extract of infant milk formula, fortified at 0.01 µg/kg of BDE-28 to BDE-183, 0.01 µg/kg of α-, β- and -γ HBCDD and 0.1 µg/kg of BDE-209, quantitative MRM transitions for α- (A), β- (B) and -γ HBCDD (C); Figure S7: GC-MS/MS (EI) chromatogram of extract of infant milk formula, fortified at 0.01 µg/kg of BDE 209, SIM 799.5 *m/z*.
