# Supplementary material for: Simultaneous Method for Selected PBDEs and HBCDDs in Foodstuffs Using Gas Chromatography—Tandem Mass Spectrometry and Liquid Chromatography—Tandem Mass Spectrometry
Source: Toxics. 2022 Dec 24;11(1):15. doi: 10.3390/toxics11010015 (PMC9862769; doi:10.3390/toxics11010015)
Supplement: Supplementary file 1 [file toxics-11-00015-s001.zip › toxics-2080269-supplementary.pdf]

toxics

SupplementaryMaterials:

Simultaneous Method for Selected PBDEs and HBCDDs in Foodstuffs Using Gas Chromatography—Tandem Mass Spectrometry—and Liquid Chromatography—Tandem Mass Spectrometry

Eva Lipičar, Danijela Fras, Nino Javernik and Helena Prosen

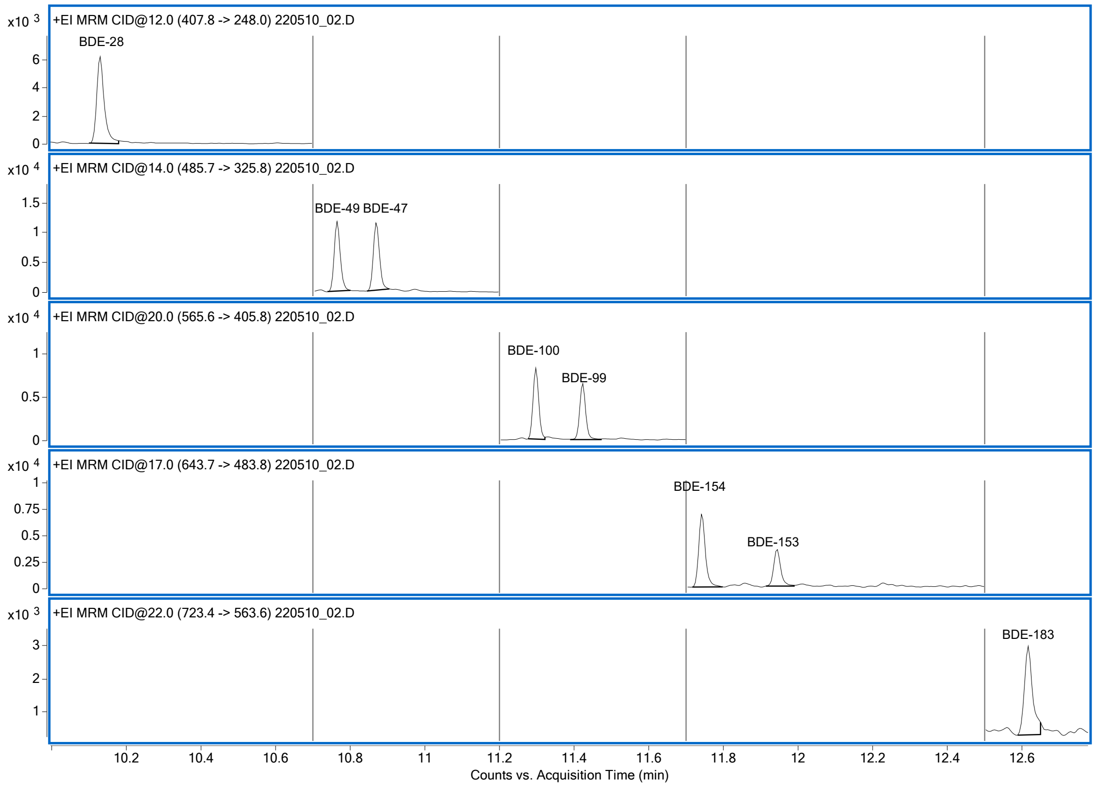

Figure S1. Chromatogram of standard mixture of PBDEs (BDE-28 to BDE-183) at 1 ng/mL, MRM transitions.

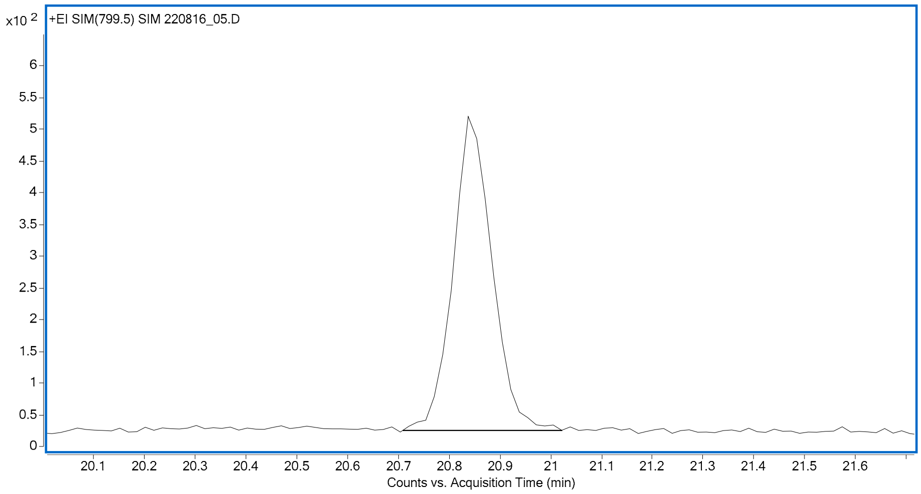

Figure S2. Chromatogram of standard solution of BDE-209 at 1 ng/mL, SIM 799.5 m/z.

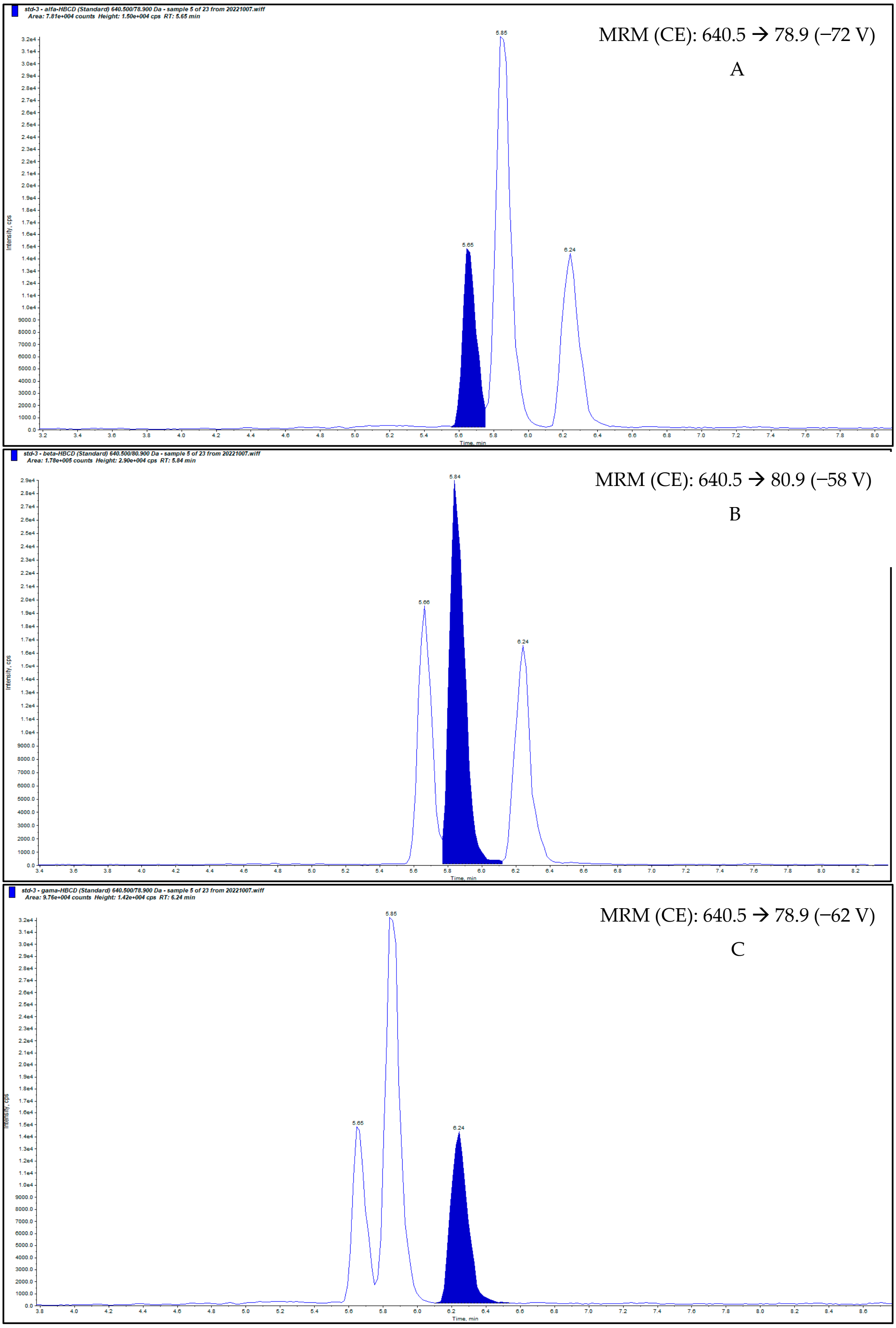

Figure S3. Chromatogram of standard mixture of α-HBCDD (A), β-HBCDD (B) and γ-HBCDD (C) at 1 ng/mL.

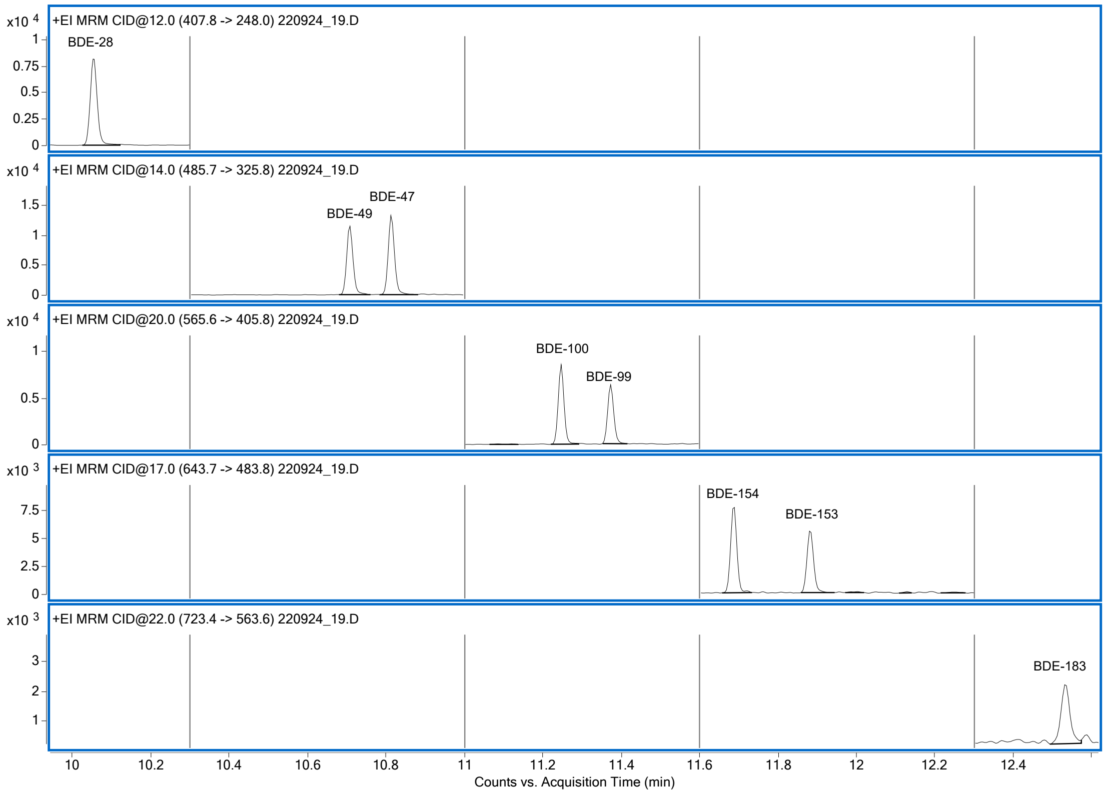

Figure S4. GC-MS/MS (EI) chromatogram of extract of infant milk formula, fortified at 0.01 μg/kg of BDE-28 to BDE-183, 0.01 μg/kg of α-, β- and -γ HBCDD and 0.1 μg/kg of BDE-209, quantitative MRM transitions for BDE-28 to BDE-183.

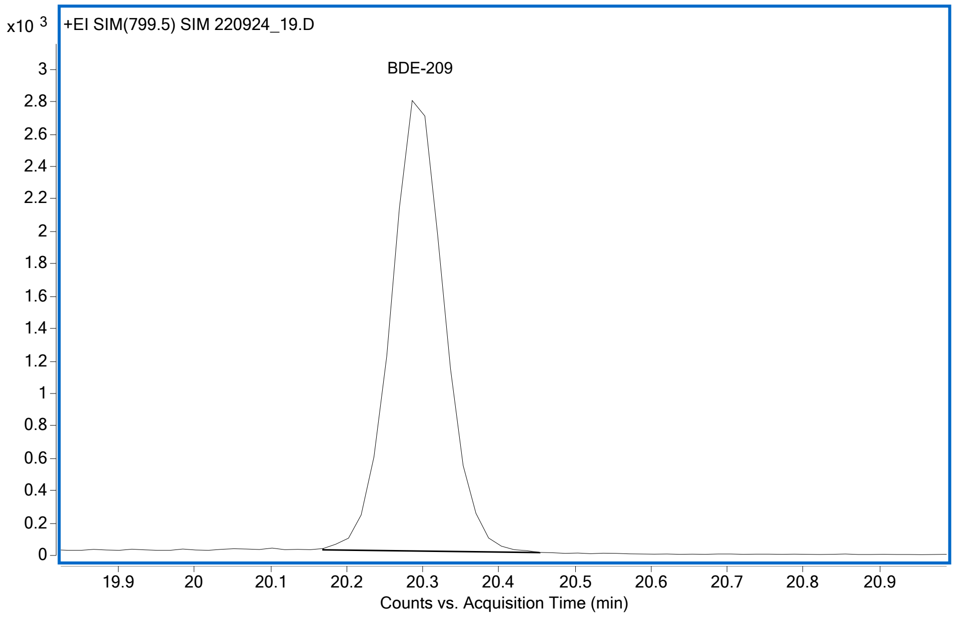

Figure S5. GC-MS/MS (EI) chromatogram of extract of infant milk formula, fortified at 0.01 μg/kg of BDE-28 to BDE-183, 0.01 μg/kg of α-,β- and -γ HBCDD and 0.1 μg/kg of BDE-209, SIM 799.5 m/z (BDE-209).

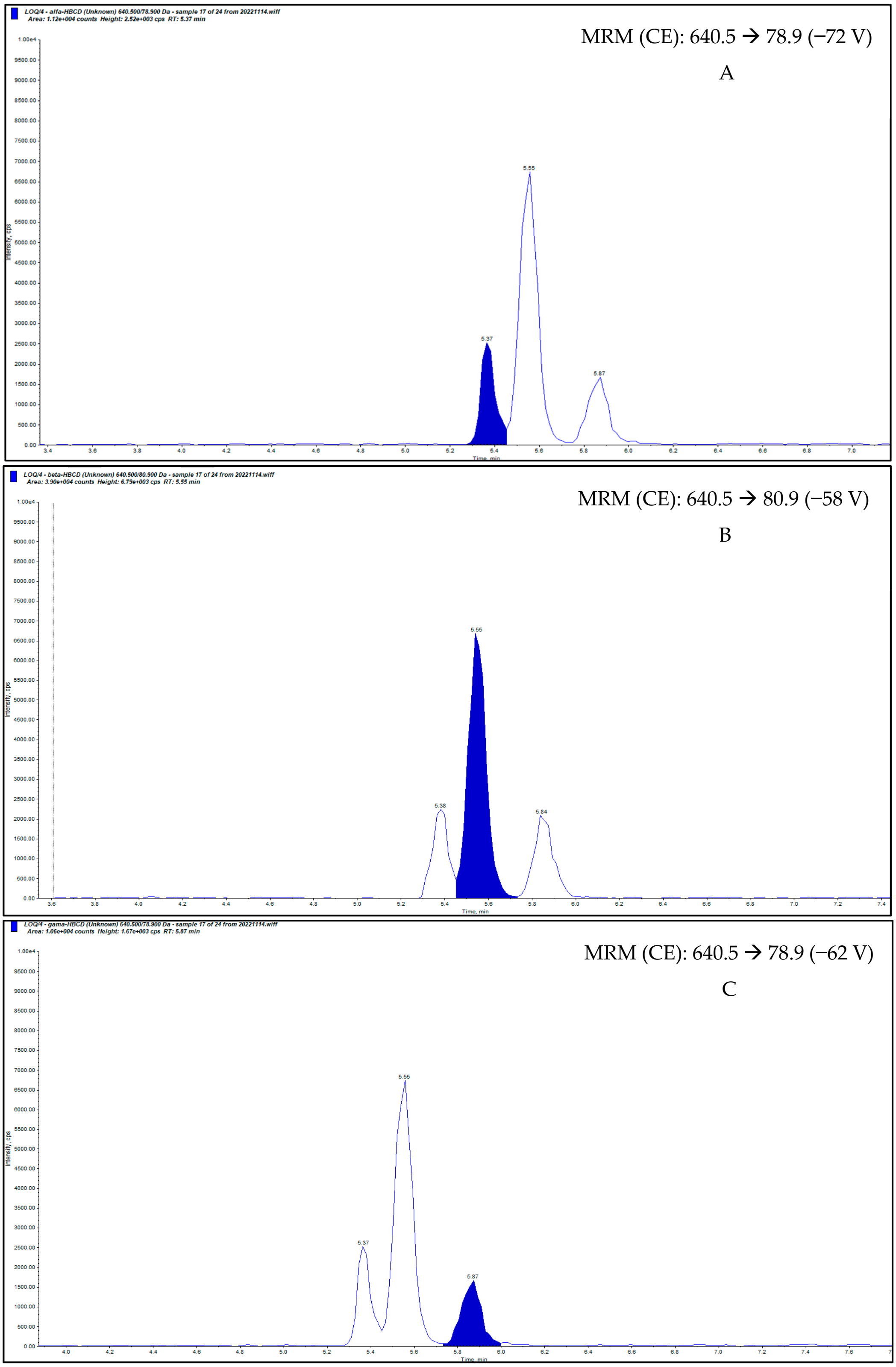

Figure S6. LC-MS/MS (ESI) chromatogram of extract of infant milk formula, fortified at 0.01 µg/kg of BDE-28 to BDE-183, 0.01 µg/kg of α-, β- and -γ HBCDD and 0.1 µg/kg of BDE-209, quantitative MRM transitions for α- (A), β- (B) and -γ HBCDD (C).

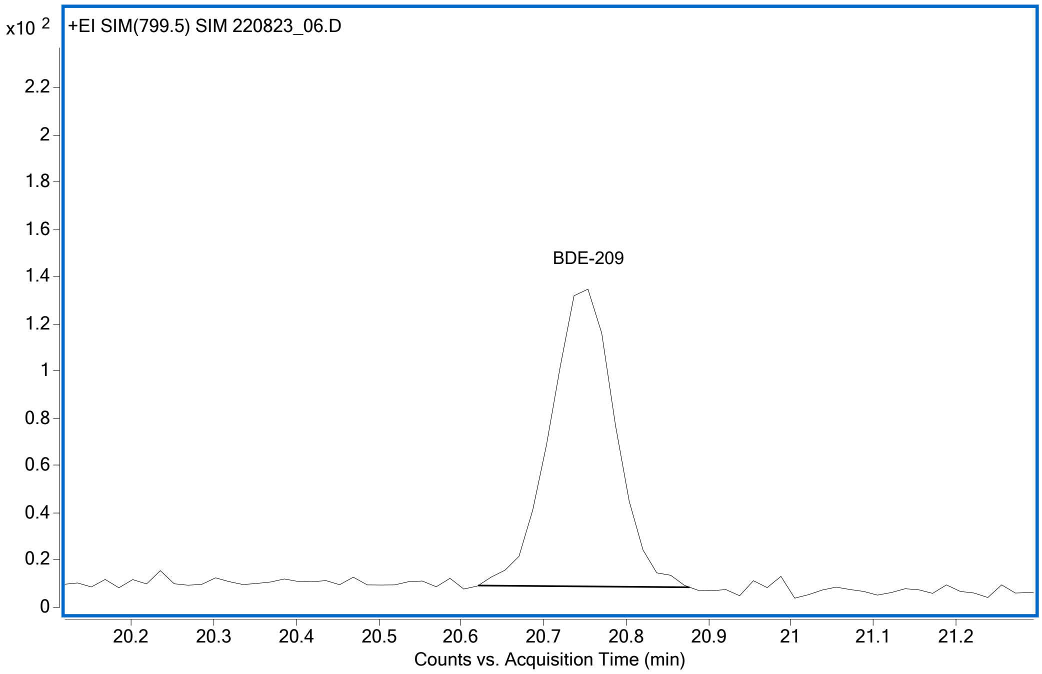

Figure S7. GC-MS/MS (EI) chromatogram of extract of infant milk formula, fortified at 0.01 μg/kg of BDE 209, SIM 799.5 m/z.
